# An Acoustic Sensor Based on Active Fiber Fabry–Pérot Microcavities

**DOI:** 10.3390/s20205760

**Published:** 2020-10-11

**Authors:** Xin-Xia Gao, Jin-Ming Cui, Ming-Zhong Ai, Yun-Feng Huang, Chuan-Feng Li, Guang-Can Guo

**Affiliations:** 1 CAS Key Laboratory of Quantum Information, University of Science and Technology of China, Hefei 230026, China; xinxia@mail.ustc.edu.cn (X.-X.G.); amz@mail.ustc.edu.cn (M.-Z.A.); hyf@ustc.edu.cn (Y.-F.H.); cfli@ustc.edu.cn (C.-F.L.); gcguo@ustc.edu.cn (G.-C.G); 2 CAS Center For Excellence in Quantum Information and Quantum Physics, University of Science and Technology of China, Hefei 230026, China

**Keywords:** acoustic sensor, fiber Fabry–Pérot cavity, micro-laser

## Abstract

We demonstrate an active acoustic sensor based on a high-finesse fiber Fabry–Pérot micro-cavity with a gain medium. The sensor is a compacted device lasing around 1535 nm by external optical pumping. The acoustic pressure acting on the sensor disturbs the emitted laser frequency, which is subsequently transformed to beat signals through a delay-arm interferometer, and directly detected by a photo-detector. In this configuration, the sensing device exhibits a high sensitivity of 2.6 V/Pa and a noise equivalent acoustic signal level of 230 $\mu \mathrm{Pa}/{\mathrm{Hz}}^{1/2}$ at a frequency of 4 kHz. Experimental results provide a wide frequency response from 100 Hz to 18 kHz. As the sensor works at communication wavelength and the output laser can be electrically tuned in the 10 nm range, a multi-sensor network can be easily constructed with the dense wavelength division multiplexing devices. Extra lasers or demodulators are unnecessary thus the proposed sensor is low cost and easy fabrication. The proposed sensor shows broad applications prospect in remote oil and gas leakage exploration, photo-acoustic spectrum detection, and sound source location.

## 1. Introduction

In many practical applications, the detection of the acoustic wave is very important, such as environmental monitoring, process control, photo-acoustic spectrum detection, and sound source location [1,2,3,4]. Electronic sound level meters based on capacitors, piezoelectric transducers, or movable coils are widely used. Especially, electret capacitive sound level meters are the most commonly used, which detects acoustic waves by measuring the dynamic deformation of a vibration film by capacitance modulation; however, this does not work well in certain situations. For example, when monitoring the operation of a high-power transformer by detecting the acoustic waves generated by partial discharges inside the transformers [5], the traditional electronic acoustic sensor could only be installed on the outside of the transformer due to the large electromagnetic field inside it, which indirectly reduces the sensor’s detection sensitivity, whereas fiber optic sensors can operate appropriately in this case. They can also perform well in other harsh environments such as deep underwater, at high temperatures, high voltage, and in small spaces due to having a compact size, light weight, high sensitivity, and an immunity to electromagnetic interference [3,6,7,8,9]. In particular, optical fiber acoustic sensors can be connected to communication cables directly, which is conducive to the integration with transmission signals. For instance, optical fiber acoustic sensors can be integrated with optical fiber networks, optical wireless communication networks, and wireless optical communication networks, which can form a remote acoustic signal monitoring network.

Several types of fiber optic acoustic sensors have been proposed [10,11,12]. The Mach–Zehnder interferometer (MZI)/Michelson interferometer (MI), fiber Bragg gratings (FBGs), and Fabry–Pérot interferometer (FPI) are three major configurations [13,14,15]. Early fiber optic sensors for acoustic signal detection were based mostly on fiber optic intrinsic interferometers such as all-fiber MI and MZI [8,16,17]. These intrinsic fiber sensors usually use single-mode fiber and coherent laser sources. The laser source is separated into two paths that have the same intensity. One arm is exposed to the acoustic signal and the other is shielded from the impact of the acoustic wave, serving as a reference arm. The reflections or the transmissions of the two laser beams are recombined to generate interference signals, which can demodulate the acoustic waves. The intrinsic fiber interferometric sensors have shown high sensitivity when a long fiber is used in the sensing arm, which indicates that they cannot achieve a single-point measurement. They are also unstable because of the drift of the source wavelength and the changes of temperature-induced optical path difference. The FBG-based sensors have also been applied in many sensing applications [18,19,20,21]. The effective refractive index of the FBG changes due to the deformation caused by the acoustic pressure (photo-elastic effect), which results in changes in the resonant wavelength. The sensing principle is typically based on the intensity modulation of the transmitted laser spectrum as the sensing element under the influence of the acoustic field; however, for practical use, the FBG-based sensor has a low sensitivity limited by the high Young module of the optical fiber material (tens of GPa), which converts the effects of high pressure applied on the grating into weak deformations, and they are insensitive to the low-frequency acoustic wave [22]. More recently, fiber optic extrinsic FPI sensors have been under development for acoustic-signal detection due to its high sensitivity. The Fabry–Pérot interferometers (FPI), which are composed of a cleaved fiber end and a reflective diaphragm, have demonstrated high sensitivity without the need long optical fiber cables [9,23,24,25]. It is one of the most versatile interferometers since its reflective diaphragm can be made of various materials such as silica [26], polymer [27], graphene [28], and silver [29]. The sensitivities of these sensors can be significantly improved by designing thinner and larger diaphragms; however, the sensor needs an extra laser system to support it, which means physically large and expensive auxiliary equipment.

Here, we propose a fiber Fabry–Pérot cavity (FFPC) based sensor with enhanced performance. The proposed sensor is an active acoustic sensor, which is essentially a laser system with much narrower linewidth and better sensitivity than a passive sensor [30,31]. It has a significantly lower cost compared to traditional sensors because it eliminates the need for narrow-linewidth tunable lasers, and only the pump source is needed, which makes the system small and more practical. Additionally, due to the large free spectral region (FSR) of the cavity, it can realize the networking of multiple sensors combined with wavelength division multiplexing devices. The micro-laser based sensor is made up of a short FFPC with gain medium, which emits a single mode 1550 nm laser with a linewidth of 3 MHz when injected with 980 nm pumping. The wavelength of the output laser is swept as the cavity length changes, thus, the external acoustic signal acting on the sensor causes the wavelength to shift. By measuring the acoustic signal via a Michelson interferometer with unequal arms, the sensing device achieves a wide frequency response from 100 Hz to 18 kHz. Experimental results exhibit a high sensitivity of 2.6 V/Pa and a noise equivalent acoustic signal level of 230 $\mu \mathrm{Pa}/{\mathrm{Hz}}^{1/2}$ at the frequency of 4 kHz. The sensor sensitivity can be further improved by using a low-noise detector and an output fiber mirror with higher transmittance, which makes it a potential platform for acoustic sensing.

## 2. Sensor Design and Measurement

**Figure 1:**
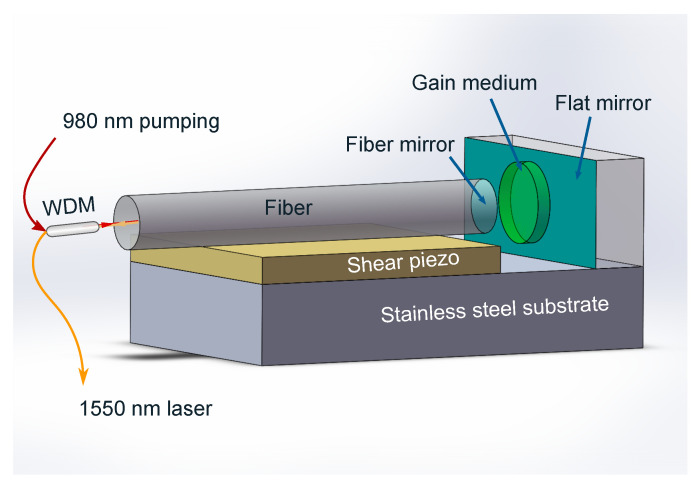
Schematic diagram of the proposed acoustic sensor based on a microcavity laser. The microcavity is a fiber Fabry–Pérot cavity composed of a concave fiber mirror and a flat mirror. An ${\mathrm{Er}}^{3+}/{\mathrm{Yb}}^{3+}$ co-doped silica film acting as the gain medium is placed inside the cavity by bonding onto the flat mirror. The fiber mirror is glued on the sheared piezo and the piezo and the flat mirror are glued together onto a stainless steel substrate. The input 980 nm pumping and output 1550 nm emission is separated by a fiber wavelength division multiplexer.

Compared to the typical FPI-based sensor, our active FFPC-based sensor is essentially a laser system with a gain medium, which can emit a tunable laser radiation around 1535 nm by a 980 nm pumping. The sensor device is a compact module with a fiber mirror and a flat mirror. Figure 1 shows our design’s plane-concave cavity for a stable cavity structure, whose concave mirror is formed by fabricating a concave on the end facet of a single-mode optical fiber.The concave spherical mirror with the curvature (ROC) of 100 μm in our cavity is fabricated by using a ${\mathrm{CO}}_{2}$ laser ablation process [32,33]. The flat mirror is a piece of a K9 glass. Both of the two mirrors are dielectric mirrors composed of ${\mathrm{Ta}}_{2}O_{5}/{\mathrm{SiO}}_{2}$ dielectric stacks based on thin-film coating technology and fabricated with ion beam sputtering, whose high reflection band is in the range of 1400–1650 nm. The reflectivity of the fiber mirror and flat mirror reaches 99.94% and 99.8% at 1550 nm and a measured finesse of the bare cavity is 2207. When an ${\mathrm{Er}}^{3+}/{\mathrm{Yb}}^{3+}$ co-doped silica film with a thickness of 35.1 μm is inserted into the cavity by bonding onto the flat mirror, which acts as the gain medium of the micro-laser, the finesse of the cavity drops to 1035 due to absorption loss of the silica film [34]. The gain peak of the doped film is around 1535 nm with ${\mathrm{Er}}^{3+}$ concentration of 1.0 wt% and ${\mathrm{Yb}}^{3+}$ concentration of 19.0 wt%. A sheared piezo is glued under the fiber mirror to control the cavity length, and they are glued onto a piece of a stainless steel substrate to form a stable FFPC. The input 980 nm pumping and output 1550 nm emission is separated by a fiber WDM.

**Figure 2:**
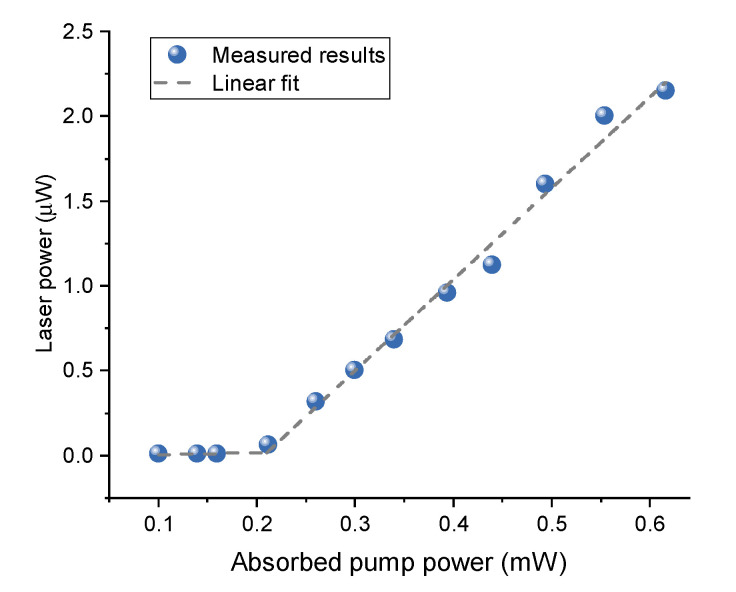
Laser threshold of the microcavity laser. The threshold is around 210 μW with the pump wavelength at 980 nm.

Figure 2 shows the laser threshold of the fiber cavity laser. When the absorbed pump power exceeds 210 μW, the lasing emission appears obviously and increases linearly with the absorbed pump power, which corresponds to a threshold of 210 μW.

For this FFPC, a with cavity length of $L_{c}$ and doped silica film thickness of $L_{d}$, the resonant wavelength approximately satisfies [35]

(1) $$n_{air}\left(L_{c}-L_{d}\right)+n_{d}L_{d}=\frac{1}{2}m\lambda ,$$

where $L_{c}$ = 46 μm, $L_{d}$ = 35.1 μm, $n_{d}=1.58,n_{air}=1$ is the refractive index of the silica film and air, respectively, and *m* is an integer, which represents different cavity modes of the resonant cavity. The compact cavity length corresponds to a large FSR of 16.5 nm (2.2 THz). When the cavity length is changed by $\Delta L_{c}$, the output laser wavelength shifts by Δλ, and the relationship between them can be expressed as

(2) $$\frac{\Delta \lambda }{\Delta L_{c}}=\frac{\lambda }{L_{c}+\left(n_{d}-1\right)L_{d}},$$

Equation (2) shows the wavelength of the microcavity laser is proportional to its cavity length; therefore, the external signal that causes the cavity length to change is obtained by detecting the change of laser wavelength. An acoustic sensor is suitable for this scenario because the acoustic pressure acting on the FFPC will lead to corresponding changes in cavity length.

**Figure 3:**
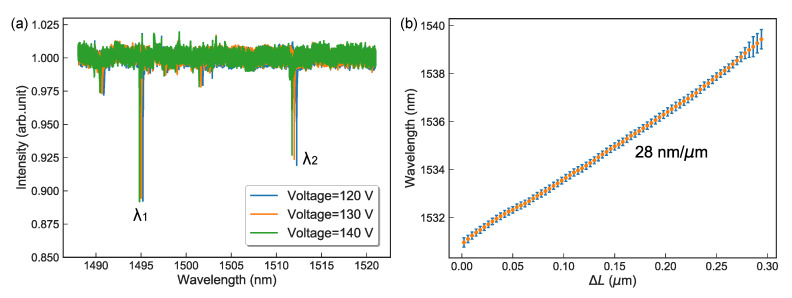
(**a**) The reflection spectrum of the cavity to calibrate the cavity length. The three reflection spectra are measured results when the piezoelectric voltage is 120, 130, and 140 V, respectively. (**b**) The wavelength shift of the output laser versus cavity length change. Resultant wavelength shifts from 1531 to 1540 nm with a 0.3 μm cavity length change, corresponding to a linear slope of 28 nm/μm.

Before acoustic sensor measurement, we measured the static cavity length displacement response of our microcavity laser. By electrically controlling the sheared piezo to change the cavity length, the tunable wavelength of the microcavity laser was recorded by an optical spectrum meter (HORIBA iHR 550). As the piezo actuators are known for their nonlinearity and hysteresis, the reflection spectrum of the cavity was measured to calibrate the cavity length. We injected a tunable laser (Toptica CTL 1500) tuning from 1488 to 1520 nm to the cavity, and the reflected laser was detected by a photodetector. As shown in Figure 3a, three reflection spectra are measured when the piezo voltage is 120, 130, and 140 V with their two resonant deeps at the wavelength of $\lambda_{1}$ and $\lambda_{2}$. By using the equation $\mathrm{FSR}=c/\lambda_{1}-c/\lambda_{2}=c/2\left(L_{c}+\left(n_{d}-1\right)L_{d}\right)$, we can calculate the accurate cavity length of 46.4425, 46.4243, and 46.3925 μm, respectively. Figure 3b shows the wavelength shift of the microcavity laser and how the cavity length changed by about 15 nm each step; with this information, we finally obtain the output laser with a total cavity length shift of 0.3 μm—the resultant wavelength-tunable range from 1531 to 1540 nm can be fitted by a linear function.

It presents a linear relationship with a change slope of 28 nm/μm, which is in agreement with the theoretical analysis that $\Delta \lambda /\Delta L_{c}=23$ nm/μm. Besides, the wavelength of the microcavity laser can be electrically tuned in 10 nm (1.3 THz) range without mode hopping; the fast tuning rate was tested as $1.6\times 10^{17}$ Hz/s. The linewidth of the laser was measured to be 3.1 MHz, corresponding to a coherence length of 66 m. These results were also found in our previous work with a microcavity laser in [34].

## 3. Experimental Setup and Principle

**Figure 4:**
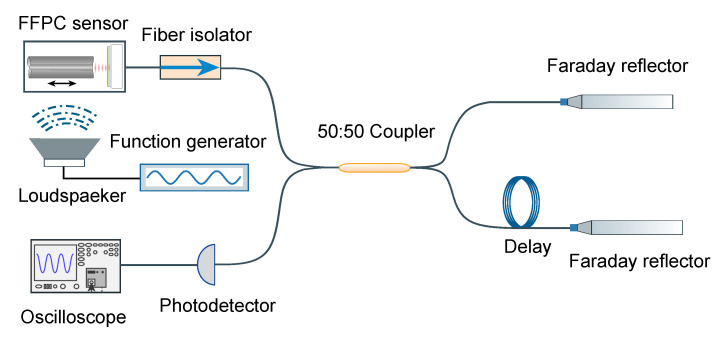
Schematic of acoustic measurement. The acoustic wave is generated by a function generator driven loudspeaker. The Michelson interferometer with unequal arms is used to measure the acoustic signal, which has a delay to produce an optical path difference between the two arms. Both arms are retro-reflected by the Faraday reflector and converged on the photodetector. The resulted interference fringes are shown on an oscilloscope.

Figure 4 shows the experimental system for acoustic sensing using our microcavity laser. The acoustic vibrations are generated by placing a loudspeaker next to the sensor, which is driven by a function generator. In this way, acoustic vibrations of different frequencies and amplitudes convert into acoustic pressure acting on the sensor element. We measured the corresponding wavelength shift based on a Michelson interferometer with unequal arms. A fiber isolator was inserted at the output of the laser in order to stabilize the output signal of the microcavity laser. The Michelson interferometer splits the output laser into two arms, one arm has a longer round trip delay *d* than the other arm. Both arms are retro-reflected by the Faraday reflector, compensating for polarization changes that are caused by the polarization-free fiber. A recombination of the two arms generates interference fringes that can be detected by a photo-diode and recorded on an oscilloscope for data processing. After the laser $I_{0}$ went through the Michelson interferometer, the interference signal *I* can be expressed as [10]

(3) $$I=I_{0}\left(1+\eta \cos\Delta \varphi \right),$$

where η is the fringe contrast, $\Delta \varphi =\varphi_{s}+\varphi_{0}$ is the total phase difference between the two arms, $\varphi_{s}$ is the phase difference induced by the optical path difference between the two arms, $\varphi_{0}$ is the phase difference including original phase difference and the phase shift induced by low frequency environmental noise. The relation between the phase difference $\varphi_{s}$ and the delay *d* can be expressed as $\varphi_{s}=4\pi nd/\lambda$, where *n* is the refractive index of the optical fiber. When the wavelength varies with the length of the cavity, the phase shift can be given by

(4) $$\Delta \varphi_{s}=\frac{4\pi nd}{\lambda^{2}}\Delta \lambda .$$

This means that the external signal modulating the cavity length to change can be obtained by detecting the phase change of the interference spectrum. In addition, Equation (4) shows the phase shift corresponding to the weak acoustic signal can be amplified by the long delay *d* (d=2 m in this work). As the laser linewidth is 3.1 MHz, corresponding to a coherence length of 66 m, the optical path difference can reach tens of meters, which can convert the extremely weak frequency change caused by the external signal into the detectable phase change.

The interference signal from the FFPC sensor is detected by a photodetector and the voltage *V* of the signal on oscilloscope converted from the optical power should be expressed as

(5) $$V=A+B\cos(\varphi_{s}+\varphi_{0}),$$

where *A* is a constant term proportional to the power of the microcavity laser source and *B* is a coefficient related to the power of the laser source and the visibility of the interference fringes. $\varphi_{s}$ is determined by the acoustic vibrations—if we give a sinusoidal acoustic signal, then $\varphi_{s}=C\sin\omega_{0}t$, where $\omega_{0}$ and *C* is the frequency and the amplitude related value of the acoustic signal, respectively. *C* represents the modulation depth at the same time. By inserting the specific form of $\varphi_{s}$ into Equation (5), we obtain

(6) $$V=A+B\cos(C\sin\omega_{0}t+\varphi_{0}).$$

Expanding this equation as a series of Bessel functions [36],

(7) $$V=A+BJ_{0}\left(C\right)\cos\varphi_{0}+B\sum_{n=-\infty ,n\neq 0}^{\infty }J_{n}\left(C\right)\cos\left(n\omega_{0}t+\varphi_{0}\right).$$

If we just focus on the high frequency component of the signal, we are going to be dealing with a mixed frequency signal of $\omega_{0}$ and high order terms of $\omega_{0}$ with the Bessel coefficient. According to the properties of Bessel function, when the modulation depth satisfies C≪1, the measured signal is dominated by the first order term $J_{1}\left(C\right)\cos\left(\omega_{0}t+\varphi_{0}\right)$, where $J_{1}\left(C\right)$ is approximately linear with the amplitude of the acoustic vibration. Thus, Equation (7) can be simplified as

(8) $$V=B^{{}^{\prime }}\cos\left(\omega_{0}t+\varphi_{0}\right),$$

which shows that the acoustic vibration frequency $\omega_{0}$ and the voltage amplitude $B^{{}^{\prime }}$ of the signal on oscilloscope is proportional to the acoustic pressure *P*,

(9) $$B^{{}^{\prime }}=kP,$$

where *k* is the sensitivity of the sensor; therefore, the frequency and the acoustic pressure of the vibration acting on the sensing element can be directly obtained from the measured interference fringes.

## 4. Results and Discussion

In this section, we present a series of measurements to evaluate the sensitivity, the frequency response, and the resolution of the microcavity laser-based sensor.

**Figure 5:**
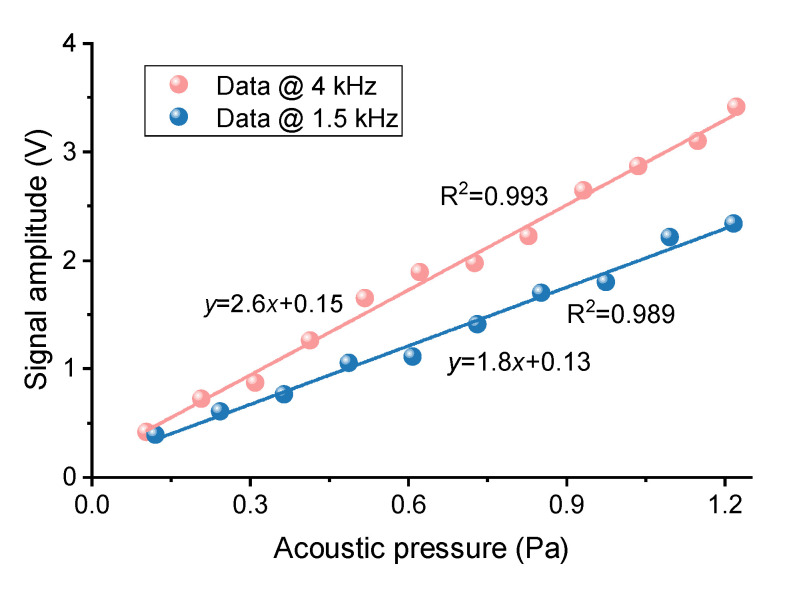
Measured signal amplitude under different acoustic pressure when the driving frequency for the loudspeaker is 1.5 and 4 kHz, corresponding to sensitivity of 1.8 and 2.6 V/Pa, respectively.

We applied sinusoidal signals with different levels of acoustic pressure at a frequency of 1.5 and 4 kHz to drive the loudspeaker, respectively. To determine the sensitivity, we used a decibel meter to calibrate the acoustic pressure. The sound intensity $I_{s}$ was converted to acoustic pressure using the relationship $I_{s}=20\log_{10}\left(P/P_{ref}\right)$ [37], where $P_{ref}=20$ μPa is the minimum acoustic pressure that humans can hear. Figure 5 shows a linear relationship between the voltage amplitude of the measured signal and the different acoustic pressure at the vibration frequency of 1.5 and 4 kHz, which shows high sensitivity coefficient of 1.8 and 2.6 V/Pa, respectively.

**Figure 6:**
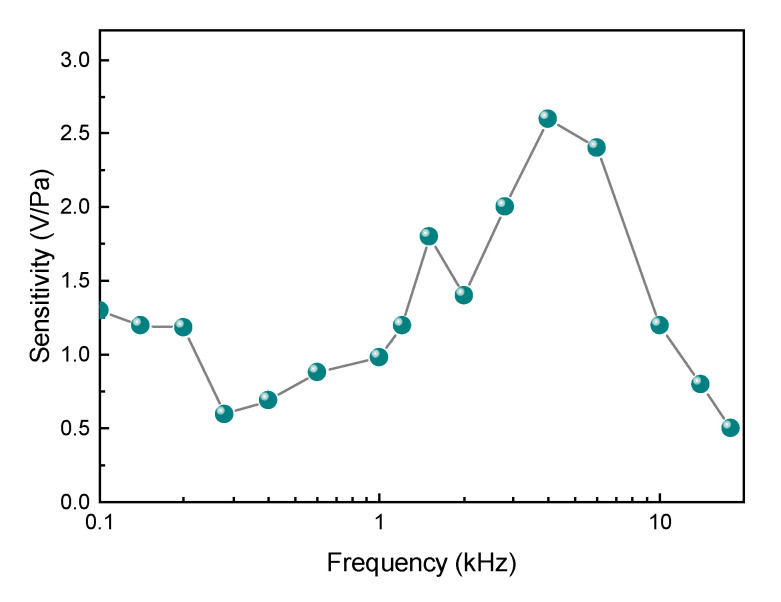
Frequency response of the acoustic sensor from 100 Hz to 18 kHz.

Figure 6 shows the sensitivity–frequency response curve. Several measurements with different driving frequencies were performed; the proposed sensor has a good frequency response over the range from 100 Hz to 18 kHz, with the maximum sensitivity at 4 kHz. The detected signal is distorted when the frequency is lower than 100 Hz, as the loudspeaker could not provide pure tone at such a low frequency.

**Figure 7:**
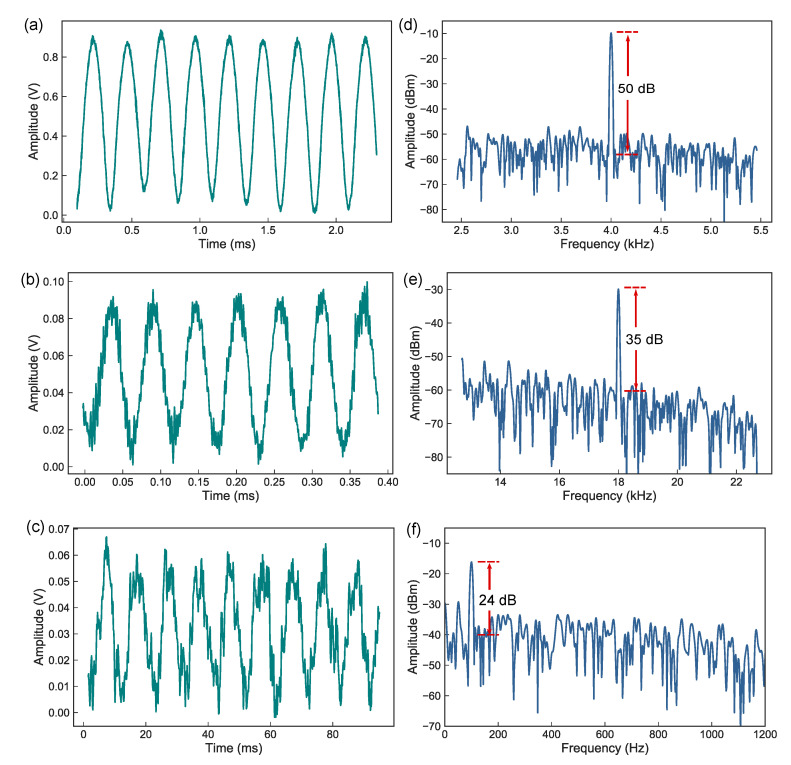
Measured signals with different frequencies shown both in time-domain and frequency-domain. (**a**,**b**,**c**) Time-domain waveform of the sensor signals when the loudspeaker is driven by sinusoidal electrical signals with frequency of 4 kHz, 18 kHz, and 100 Hz, respectively; (**d**,**e**,**f**) the corresponding frequency-domain spectra of (**a**,**b**,**c**).

The measured signals in both time-domain and frequency-domain are presented in Figure 7 by applying three different frequency sinusoidal signals at 4 kHz, 18 kHz, and 100 Hz on the sensor. Figure 7a–c show the time-domain sine-like waveforms; Figure 7d–f show the corresponding frequency-domain spectra, whose peak values are consistent with the driven frequencies of the loudspeaker. The frequency-domain signal was recorded by a spectrum analyzer; Figure 7d,e show the noise floor about −60 dBm for a 14.66 Hz resolution bandwidth (RBW); Figure 7f shows the noise floor about −40 dBm for a 7.82 Hz RBW. The signal-to-noise ratios (SNR) of the measured signal are 50, 35, and 24 dB, respectively. Its noise equivalent acoustic signal level can be calculated by [38]

(10) $$\delta P=\frac{P_{ref}}{\sqrt{\mathrm{RBW}\times \mathrm{SNR}}},$$

where $P_{ref}=288$ mPa is the referenced acoustic pressure applied to the sensor, resulting δP=230 $\mu \mathrm{Pa}/{\mathrm{Hz}}^{1/2}$ at the frequency of 4 kHz, which means the minimum detectable dynamic acoustic pressure value of the FFPC sensing system at 4 kHz frequency is 230 μPa, which can be significantly improved by using a low-noise detector and an output fiber mirror with higher transmittance.

The change in air refractive index Δn affected by acoustic pressure fluctuations *p* can be presented as follows [39]:

(11) $$\Delta n\approx p\left(n_{0}-1\right)/\left(\gamma p_{0}\right),$$

where $n_{0}=1.00027$ is the air refractive index under static conditions, $p_{0}=10^{5}$ Pa is the static atmospheric pressure, γ=1.40 is the heat capacity ratio (or ratio of specific heat) of air. By applying an acoustic signal of 1.2 Pa, the corresponding refractive index difference is $\Delta n=2.4\times 10^{-9}$. From Equation (1), it can be deduced that Δλ/Δn=0.255 μm, then the acoustic pressure of 1.2 Pa causes the wavelength shift $\Delta \lambda =6\times 10^{-16}$ m; however, in the experiment, the amplitude of the measured signal was 3.4 V when the acoustic pressure was 1.2 Pa. As the responsivity of the photodetector at 1535 nm is 1.02 A/W with a high gain coefficient of $7.5\times 10^{5}$ V/A, this would convert the input laser intensity $I_{0}=10$ μW to 7.5 V. Thus, a phase difference shift of about 0.456 rad is calculated from Equation (8), which corresponds to a wavelength shift of $3\times 10^{-14}$—100 times as large as the wavelength shift caused by the refractive index change of the air in the cavity—therefore, the main interaction mechanism is not the refractive index change of the air, but maybe the fluctuation of the bare fiber or or the instability of the mechanical structure of the system, resulting the cavity length to change, which could be further investigated in future research. As the phase difference of 2π/10 is easy to detect in the Michelson interferometer, we obtain the corresponding wavelength shift of $3.8\times 10^{-14}$ m by Equation (4), resulting in a cavity length change of 1.4 pm according to $\Delta \lambda /\Delta L_{c}=28$ nm/μm measured above. It means that a change in cavity length on the pm scale can be detected, which is due to the short length of the microcavity, the laser wavelength changes by 16.5 nm when the cavity length varies by half a wavelength. In the case of the same deformation, this sensor is much more sensitive than the ordinary MIZ/MI sensors, because they directly measure the change of fiber length, which requires nearly half wavelength to generate an obvious signal, while the cavity length change of pm scale is detectable in our microcavity. For typical FPI sensors, they usually have a low finesse of about 10 [28], which corresponds to the much wider linewidth of the reflective spectrum without sufficient coherence length for converting the extremely weak frequency change into the detectable phase change. Therefore, it’s a promising device in acoustic sensor.

In the actual application process, the influence of the environment noise causes the arm length difference of the interferometer to fluctuate, thus generating additional phase noise; therefore, the interferometer can be well packaged to improve its anti-interference ability to the environment noise.

## 5. Conclusions

In summary, we have designed and fabricated an active acoustic sensor based on a fiber cavity laser, which is a low-cost device because it eliminates the need for commercial lasers or demodulators. Thus, it is easier to make the system small and has stronger practicality. The sensing device provides a wide frequency response from 100 Hz to 18 kHz and exhibits a high sensitivity of 2.6 V/Pa with a noise equivalent acoustic signal level of 230 $\mu \mathrm{Pa}/{\mathrm{Hz}}^{1/2}$ at the frequency of 4 kHz, which can be significantly improved by using a low-noise detector and an output fiber mirror with a higher transmittance. Additionally, due to the large tunable range of the micro-laser, it can realize the networking of multiple sensors combined with wavelength division multiplexing devices, which makes it a good candidate in remote oil and gas leakage exploration, photo-acoustic spectrum detection, and sound source location.
